# Challenges in Organ Transplantation

**DOI:** 10.5041/RMMJ.10049

**Published:** 2011-04-30

**Authors:** Rafael Beyar

**Affiliations:** Director, Rambam Health Care Campus, Haifa, Israel; Professor of Medicine & Biomedical Engineering, Women’s Division – Dr Phillip & Sarah Gotlieb Chair, Technion, Haifa, Israel; Chairman of the Steering Committee of the Israel National Transplantation center, Ministry of Health, Tel Aviv, Israel

**Keywords:** organ transplantation, Israel National Transplantation Committee, brain and respiratory death

## Abstract

Organ transplantation has progressed tremendously with improvements in surgical methods, organ preservation, and pharmaco-immunologic therapies and has become a critical pathway in the management of severe organ failure worldwide. The major sources of organs are deceased donors after brain death; however, a substantial number of organs come from live donations, and a significant number can also be obtained from non-heart-beating donors. Yet, despite progress in medical, pharmacologic, and surgical techniques, the shortage of organs is a worldwide problem that needs to be addressed internationally at the highest possible levels. This particular field involves medical ethics, religion, and society behavior and beliefs. Some of the critical ethical issues that require aggressive interference are organ trafficking, payments for organs, and the delicate balance in live donations between the benefit to the recipient and the possible harm to the donor and others. A major issue in organ transplantation is the definition of death and particularly brain death. Another major critical factor is the internal tendency of a specific society to donate organs. In the review below, we will discuss the various challenges that face organ donation worldwide, and particularly in Israel, and some proposed mechanisms to overcome this difficulty.

## PREFACE

Organ transplantation has a key role in medicine worldwide and has become an essential treatment modality in saving and prolonging lives in a wide variety of clinical conditions. Kidney, heart, liver, lung, and pancreas are among the vital organs that are routinely used for transplantation, but many other organs that draw less public attention such as small bowel, skin, ligaments, bones, and cornea are used in various clinical conditions to provide temporary or permanent relief for various clinical conditions. In general, organ transplantation saves lives, prolongs survival, and increases the quality of life. Kidney transplantation has been proven to have a survival advantage over hemodialysis, accompanied by a marked increase in the quality of life. In general, organ implantation is co-ordinated via regional or national allocation programs, which set up the priorities for organ allocation and provide the essential logistics and laboratory support for the transplantation process.^1^,^2^ These organs can be preserved for a relatively short period of time, and therefore mechanisms for immediate organ allocation, once a donor is identified, are critical.

Organ transplantation is one of the most complex procedures in medicine for several reasons. First and foremost, it involves dealing with the medical aspects of the recipient patient in parallel to dealing with a matched donor in case of a living donor or his family for a deceased donor. Whether involving a deceased or a living donor, the ethical rules that wrap the process of organ transplantation are complex and often convoluted by ethical and religious nuances. Ethical issues with the timely and unequivocal definition of death are among the most debatable and complex dilemmas in medicine,^3^–^5^ and the public opinion is often skewed by religious and cultural influences and ethical standards that vary between different cultures and religions. On top of that, the field of transplantations is faced with a worldwide shortage of organs,^6^,^7^ and this mandates the need to guard the ethical standard of medical priorities for those patients that depend on the transplantation to save their lives.

In this review, I will discuss the major dilemmas that we face in Israel and worldwide regarding organ transplantation.

## SHORTAGE OF ORGANS

The shortage of organs is a major problem worldwide.^6^,^7^ There are many more patients awaiting transplantation than there are organ donors. The improvement in medical and surgical techniques that enable transplantation to take place in cases we would not consider a decade ago has not been matched with a parallel increase in the availability of organs for transplantation, and the problem of organ shortage has become more profound. In 1999, some 40,000 Americans were on the waiting list for kidney transplantation according to the Scientific Registry of Transplant Recipients. By 2009, the list had grown to nearly 83,000 people, whereas only 16,500 people received a transplant.^8^ In Israel, the number on the waiting list for kidney donors has increased from 490 in 2006 to 690 in 2010, while the number of kidney transplants from deceased donors decreased from 87 to 65.^9^ At the same time there was an increase in live kidney donations from 54 to 78. Thus, taking into account transplants from both deceased and living donors, there is only about one donor for every five potential recipients, both in Israel and the USA. Similar shortage is also present for other organs. In Israel, 151, 133, and 66 patients were waiting for liver, heart, and lungs, respectively, whereas only 46, 32, and 11 transplants were performed in 2010.

The shortage of organ donors is multifactorial. In general, the number of potential donors that meet the criteria of a brain death diagnosis is far greater than the number of utilized donors where transplantation took place. The difference between these numbers is due to medical and logistic factors, the ability to determine brain death, and cultural and religious factors that affect the willingness of the population to donate organs. As a result of these factors, there is a large variability in organ donation rates among countries,^10^ and, therefore, the waiting time for transplantation is largely variable.

Shortage of organs should be analyzed separately for living and deceased donors. For deceased donor programs the most important factor is the availability of a sound national or regional transplantation program that meets international standards. According to the World Health Organization (WHO) criteria, such a program should be present in each country, so that it becomes self-sufficient over time with respect to its population organ needs.^11^ An important factor is the cultural compliance and general consent of the society to organ donations. There are many and variable ethical and religious issues related to organ donation. While in all major religions organ donation is encouraged in order to save lives, there may be huge differences in the practical approaches to the donation process among different factions even within the same religion.

## THE DEFINITION OF DEATH AND THE ISRAELI LAW FOR BRAIN AND RESPIRATORY DEATH

The definition of death is a critical step in deceased donor transplantations and often the most problematic and emotional stage. The discussion about the definition of death has involved not only the medical community, ethicists, and philosophers but also almost all the religious leaders. While the different religions may have different attitudes towards the definition of death, all are in agreement that the brain is the critical organ in the definition of death, and brain death equals death. All agree that it is mandatory to provide sound evidence that the brain is irreversibly dead. While, in general, the public accepts the medical judgment in the definition of death, there are occasional cases where a family does not agree with the diagnosis of brain death by the medical team. This may result from the personal beliefs, emotions, and distrust of the family in the medical system. Often, in critical moments of imminent death, several families, not necessarily religious, seek advice from religious authorities and submit to their judgments. It is therefore clear that trust between the medical community and the public, through its religious leaders, is a critical element in this complex process of accepting brain death and agreeing to organ donation when appropriate.

A recent law on the definition of brain death was passed in Israel in 2008 and has been in effect since the middle of 2009.^12^ The law involves medical, ethical, and religious aspects of death and defines strict rules as to how and by whom the diagnosis of brain death should be confirmed. The law mandates the use of objective diagnostic tests such as transcranial Doppler, brain angiography, and single photon emission computed tomography (SPECT) imaging and also sets an uncompromised requirement for the apnea test despite many pitfalls that may exist with this study.^13^ The physicians are required to provide evidence by mandatory ancillary tests of no brain activity, no blood-flow to the brain, and no respiratory drive. One of the major reasons for the new law was to ensure standardization of the procedure of brain death diagnosis across all hospitals in Israel. Therefore, the law allows very limited clinical judgment in brain death diagnosis and mandates confirmatory ancillary tests.

It was hypothesized that the new law, by providing standardization of brain death criteria which are not open to interpretation, would relieve the tension within the community with respect to the definition of brain death and would increase the trust between the medical community and the general public, including the religious sections. It was therefore suggested that the law would increase the tendency of the public to consent to organ donation and would alleviate the shortage of organs.

As the law was implemented in the middle of 2009, comparing transplantation data during 2010 to parallel data during 2008 provides a reasonable estimate for the immediate effect of the law (Table 1).

As can be seen from the data in Table 1, what actually happened in the first year after the implementation of the law was in opposition to the hypothesis that the law would convince more people to donate. The numbers of consents to donate and actual organ donations have decreased substantially, and the number of organs transplanted was therefore reduced. With respect to kidney donations, the sharp decrease in deceased kidney transplantations was partially balanced by an increase in live donor kidney transplantations.

The factors that led to such a devastating outcome were the strict requirements for confirmatory tests, without mechanisms to provide an alternative pathway in cases where these tests are meaningless or cannot be performed for medical reasons, and not allowing the professional committees to decide about ancillary tests. Sometimes a delay in the definition of brain death in itself has led to the loss of the patient organs, as multi-organ failure occurred before transplantation could be resumed. So, while the law provided a standard definition of death across the country, it also prevented the definition of brain death in a timely manner in a significant number of patients, as well as created a harmful burden on transplantations in the first year of its effect. We have also observed that the negative approach to organ donation was enhanced in some portions of the public rather than decreased. Another possible explanation for the drastic reduction in donations in the first year after the implementation of the law may be the incomplete organization of the medical community to the new practice of brain death definitions, i.e. proper training to all physicians, availability of ancillary tests, and expert teams in all hospitals, etc. Therefore, a greater efficiency in conducting ancillary tests in the process of brain death diagnosis in a timely and professional manner may improve the results over time, but this remains to be seen.

It is my understanding, based on the above, that while the strict standard criteria that are now fully imposed by law create more robust standardization among hospitals in Israel, a mechanism for confirming brain death in those patients where some ancillary tests are inadequate or impossible must be implemented. There are more than a few examples where possible donors, who had expressed their wish to donate organs during their lives by signing a donor card, could not be diagnosed as brain-dead because of the barrier of the law and ended up dying without fulfilling their request (Table 2). Therefore, it is suggested that the institutional committees for determination of brain death should be allowed to decide whether and when to use ancillary testing. Thus, instead of being *mandatory* in all cases, these tests should be *indicated* in circumstances where one or more of the brain stem tests, e.g. apnea test, cannot be performed.

While the requirement for definition of brain death is global and well agreed upon, there is no uniformity in methods and responsibilities among countries and even between hospitals within the same country.^14^,^15^ From a legal perspective, each country or state has its legal regulations for death. On the basis of these regulations, each hospital establishes criteria for the determination of brain death. Subsequently, a large variability in the determination of brain death between and within individual hospitals has been reported in American and European hospitals.^14^,^15^

## ETHICAL RULES FOR LIVING DONORS

Living donor donations are widely used worldwide, and the numbers are constantly increasing. According to recent publications, 27,000 living donor kidney and 2,000 living donor liver transplants are performed worldwide annually.^16^,^17^ The shortage of deceased donor organs led to a steady increase in live donors over the last years.

The ethical rules for live donation are different than those for deceased donors, but what is common to both is the extensive attention to the act of organ donation by ethicists, religions, and the medical communities. The majority of live organ donations are kidney transplants, followed by partial liver and partial lung transplants. The main ethical principle in live donations is to cause little or no harm to the donor. Organ donations between family members are well accepted and valued by society. It is also accepted that altruistic donations, those with a pure and non-financial motivation to help a patient suffering, are a noble thing. However, any donation which is associated with financial payment for the organ is generally unacceptable. While arguments are voiced that patients may have the rights over their bodies and they can “sell” organs as they wish, it is widely accepted that such practice is unethical and should be banned. Organ trafficking has been and continues to be a major problem in the world. Modern societies worldwide are now strictly against organ trafficking, and international actions are taken to prevent such cases.

In 2008, the Declaration of Istanbul on Organ Trafficking and Transplant Tourism, the European Parliament, and the Asian Taskforce on Organ Trafficking each issued formal statements urging member states to define conditions in which reimbursement can be granted.^18^ A clear distinction is made between the acceptable practice of reimbursement of legitimate expenses incurred due to the transplant process and payment resulting in illegal financial gain. In Israel, according to a recent law on organ transplantation that is in effect since 2008, direct payments to donors from another source or from insurance are now illegal.^19^ At the same time the law allows for compensation of the direct expenses of organ donation incurred by the donor and also adjustment of his medical insurance benefit to his new more liable condition. In addition, this new law also prioritizes organ donations to persons and families who have committed to organ donation during their lives (signing the ADI card – the Israel organ donor card) or to those who have donated organs in the past. This is a unique law that creates a formal national mechanism for compensation from society to organ donors for expenses incurred.

Following a recent case in Israel, where family members have not consented to organ donation from a deceased patient who possessed a donor card (ADI), a public discussion on the legal binding power of the donor card has emerged. The current legal status is that the donor card is not a binding contract. In the overall evaluation of a possible effect of such a legal change, it should be understood that only few cases have been reported where a wish of an individual to donate organs, as expressed by a signed card, is not respected by the family. Therefore, providing a legal power to a donor card may interfere with the signing process and have a negative effect on the attitude of the public to organ donation.

A comprehensive overview of legislation and practices of reimbursement for living organ donors is provided by Sickand et al.^20^ There are at least 20 countries where living donor reimbursement exists in various forms. Many programs have recently been implemented in various countries; however, most living organ donors worldwide lack organized programs to defray the costs of the donation process. The concept of a central body that has the authority and structure to compensate live donors as well as families of deceased donors has been proposed and is legally supported in various countries, including Israel. Such a body can allocate reimbursement funds for the medical and other expenses associated with transplantation and can provide a mechanism by which society takes care of those individuals who gave to society one of the highest values in human ethics – life.

## EFFICIENCY OF THE TRANSPLANTATION NETWORK PROGRAM

The efficiency of a national or regional program depends on its ability to identity potential donors, to track their condition, to be in contact with the patient’s families, to follow closely all the confirmatory tests and actions required to diagnose brain death, and to provide an accurate system for matching and allocation of the organs. The WHO has set criteria and mechanisms to track the efficiency of the different steps in organ donation.^11^ In Israel, once a possible donor is identified, he is followed by a co-ordinator of the National Transplant Program who is in charge of all the processes from this point and on. Definition of brain death is done by a special committee that has undergone formal mandatory training, in compliance with the new law on brain and respiratory death.^12^ Organ matching and allocations are done through a national database with strict criteria accompanied by extensive testing and validation processes. An efficient harvesting and implantation system is obviously the highlight of the transplantation process.

Organ donation activity is reviewed by the Israeli Transplantation Center on a yearly basis, to track the completeness and appropriateness of the complex process of possible deceased donor identification and handling (Donor Action). In Table 2 the nation-wide data for 2010 Donor Action are provided. The database is based on 700 patients with brain damage who were reviewed retrospectively. The list was reduced successively towards a total of 54 actual and 52 utilized donors. While this is a natural process and occurs with all programs, there are some unique observations that should be considered. In a relatively large number of patients the families did not agree to determine brain death or did not accept the medical diagnosis of brain death when it was reported to them by the medical team. In some cases brain death could not be determined due to inability to perform apnea test, or due to logistic problems in conducting the mandatory confirmatory tests. The overall consent of the families was 50%. It is clear that a higher consent rate at an earlier phase of the process will lead to more potential donors becoming utilized donors.

The processes when interacting with families of patients in critical conditions approaching death are complex and distressing and are dependent on attitudes, beliefs, and religions. Often, family decisions are guided and modified by religious authorities. In Israel, as in other countries, the rabbi, imam, or the priest is often involved at various stages of these complex decisions at a very difficult time for the families.

## SPECIAL PROGRAMS

Special programs have been implemented to enhance the efficiency and increase the availability of organs. For living kidney donations there is often an incompatibility mismatch between donor–recipient pairs that prevents the transplant. Both kidney paired donation and desensitization are optional solutions for these patients. Kidney paired donation is a program in which kidney donor–recipient pairs who are not compatible to each other participate in a wider matching program, thus optimizing the use of kidneys in these conditions.^21^,^22^ Such a program matches a living donor with a compatible recipient in a tag-team approach among potential donor–recipient pairs and can achieve compatible transplant combinations. Desensitization therapies have also been used to achieve transplantation from an incompatible donor; however, such procedures are costly and may have associated complications and inferior long-term outcomes.^23^,^24^

Programs for non-heart-beating-donors (NHBD) exist for at least 10 countries in Europe.^25^ Between 2000 and 2008 a total of 4,908 organs were implanted, with the vast majority being kidney transplants, but also lung, liver, and pancreas transplants were carried out. Therefore, such programs can increase the availability of organs for transplantation. Organs from NHBD are more difficult to harvest, as it requires special attention and an immediate response set-up. Preservation of organs after death due to cardiac arrest is limited in time, and the preservation system must be initiated early in order to allow harvesting and transplantation of organs. When a patient dies with cardiac arrest, the other vital organs can be preserved, but for a limited time only, until harvesting and implantation can take place. Actions to preserve the organs involve inserting special cannulas that can perfuse the kidneys or other organs with the adequate preservation solutions, until consent is obtained from the family, and until the surgery can take place. Obviously, family consent is mandatory in most countries before harvesting can take place; however, special cannulas must be inserted promptly during cardiopulmonary resuscitation, before consent is given. This can be viewed as a temporary organ preservation act until the family and patient’s past requests can be validated. This assures that the rights of the patient or the family to agree to organ donation can be preserved until they can be reached and consent sought.

While such programs may require a special set-up and expertise, they can increase the availability of organs for donations by 10%–30% if done properly.^26^,^27^ In Israel such programs are not implemented yet, although planning is underway.

Immunosuppressive therapy, preventing organ rejection, has been the landmark in organ transplantation, with calcineurin inhibitors (CNI) being the backbone of this treatment. Nevertheless, major adverse events and persistent risk of chronic graft rejection continue to be a challenge to transplantation. Development of new agents with modern techniques to monitor immunosuppressant activity has made significant progress.^28^ The mammalian target of rapamycin (mTOR) inhibitors sirolimus and everolimus involve a class of drugs suppressing T cell proliferation and reducing tumor growth. In solid-organ transplantation, the combination of a CNI and an mTOR-inhibitor is a potent immunosuppressive therapy that effectively prevents the incidence of acute rejection, although the potential nephrotoxic impact must be considered in the longer term. There is no doubt that increased understanding of immune responses to transplantation, with development of new therapeutic regimens, will lead to more potent and less risky adverse event profile and will continue to improve both the short- and long-term outcome of organ transplantation.

Presumed consent for organ transplantation is legislated in several countries. It has been claimed that presumed consent may increase the rate of deceased organ transplantations. Rithalia et al.^29^ have reviewed five studies comparing donation rates before and after the introduction of legislation for presumed consent, eight studies comparing donation rates in countries with and without presumed consent systems, and 13 surveys of public and professional attitudes to presumed consent. The authors conclude that presumed consent is associated with increased organ donation rates; however, it is unlikely to be the sole explanation for the variation in organ donation rates between countries. It cannot be inferred that the introduction of presumed consent legislation per se will lead to an increase in organ donation rates, as it depends on many other factors, such as the availability of potential donors, infrastructure for transplantation, quality of health care, and underlying public attitudes.

Allocation of organs which depend on the type of organ transplanted has also been challenged recently. Typically, cadaveric kidney allocation has been done based on waiting time, while liver, lung, and heart allocation often depends on the urgency of the transplantation. The current allocation algorithm does not account for differences in potential survival of recipients and donated organs but focuses on waiting time rather than appropriately weighted medical factors. It allows kidneys with very short potential survival to be distributed to candidates who are expected to survive for a long time, and, conversely, leads to reduced organ survival when a high potential survival kidney is allocated to a patient with a short life expectancy. Recently, the Organ Procurement and Transplantation Network (OPTN) released a proposed concept for the allocation of kidneys from deceased donors that uses the Kidney Donor Profile Index (KDPI), ranking each kidney according to the length of time that it would be expected to function^30^ A method for survival matching between the transplanted kidney and the patient based on the KDPI is proposed. This new concept, trying to optimize the expected survival time of organs and patients, makes a lot of sense as it can generate a much more biologically plausible condition and as it can make more efficient use of the very scarce supply of donor organs. We will have to wait and see how society adopts these concepts.

## INTERNATIONAL COLLABORATION

International collaborations are required to optimize the process of organ matching and donation and to generate solutions in unique situations, where organs are urgently exchanged between countries to save a critically ill recipient and in cases where matching cannot be obtained within the same country and the available organ can be used elsewhere in the world. For international patients seeking transplantation, rules exist in certain countries where a certain number of foreign patients can be included. For example, such programs exist in California (5% can be from another country) and in some European countries. While trafficking and selling organs is banned by international standards as a valid method for organ transplantation, it is still a problem in certain countries. Collaboration programs between transplantation and health care centers are encouraged, and as an example Israel has already signed a contract with the Eurotransplant International Foundation.^31^ In fact, a donor liver was recently shipped from Israel to a child recipient within Europe, as no match could be found among Israeli patients and a sick child was successfully transplanted in Germany. Such examples exist worldwide and are encouraged.

While such international exchange collaboration programs can solve some individual acute or subacute problems, they are not a mechanism to balance the variability in organ donations between countries. It is widely agreed that a shortage of organs in a certain country cannot be corrected through transplantation programs elsewhere in the world. It is the responsibility of the health care system within each country, together with its social ethicists and religious leaders, to assure that an efficient organ transplantation program is implemented and that the public is educated towards donating organs and saving lives.

In Israel, a very organized and well defined program is present at the national level; however, the apparent shortage of organs is in part due to the public’s relatively low acceptance of organ donations. Intensive programs to enhance the public awareness towards organ transplantation and to increase the consent rate to organ donations are now being carried out in Israel. It includes national public awareness programs that involve all communication media, discussions with religious and community leaders, and comprehensive research and surveys to understand the multiple parameters that affect public opinion with respect to organ donation.

## THE FUTURE OF TRANSPLANTATION MEDICINE

The surgical expertise, logistics, biology, and pharmacology of organ transplantation are constantly progressing and continue to impact this field. Organ preservation is becoming more efficient and is associated with less injury to the transplanted organs. We are now able to transplant organs which are less optimal and to older and sicker patients. With the excellent medical and surgical expertise and progress in immunology and pharmacology, the main limitation is public awareness and the general consent of society to organ donations. It is a complex problem that involves intense ethical and religious discussions, but it is up to the societies across the world to be convinced that this is the only way today to save lives and increase the quality of lives in these devastated groups of patients who need vital organ donations.

As to very futuristic ideas of being able to engineer organs and use transplants from animals^32^ this is still years and maybe decades away from any possible solution. As an alternative to heart transplantation, ventricular assist devices and artificial hearts are being used today as a definite therapeutic mode and have been shown to prolong lives as compared to medical therapy alone. However, no artificial organ, kidney, or heart can be comparable, in providing the span or the quality of life, to a successfully transplanted organ.

## SUMMARY

The world of transplantation has gone through major changes and progress over many years, with superb methods to enhance our organ preservation and surgical and immunologicpharmacologic therapeutic abilities. However, the major burden on transplantation across the world is shortage of organs, which critically depends on the agreement of the public to organ transplantation. As a global society we should ban organ trafficking and organ selling worldwide and act against this phenomenon. At the same time, we should continue our efforts to optimize our regional and national organ transplantation programs, increase public awareness of organ donation, encourage public opinion and religious leaders towards acceptance, and educate our medical community, to reach a goal where the majority of eligible patients consent to organ donation.

## Figures and Tables

**Table 1:** Comparison of transplantations before and after the “Law for Brain and Respiratory Death” that was implemented during 2009.

	**2008 – before law**	**2010 – after law**
Consented to donation (pts)	72	60
Organs transplanted	280	238
Deceased kidney donations	100	65
Live kidney donations	56	78

**Table 2:** National data for critical pathway on deceased donation based on data from Donor Action of the National Transplant Center in Israel, 2010.

Total patients with severe brain damage studied	700
Potential donors (suspected to fulfill brain death criteria)	186
Potential donors reported to the Israeli Transplant Center	186
Brain death not determined as per family request	33
Brain death not determined for logistic problems	6
Brain death determined, family does not accept death	24
Brain death could not be determined for medical reasons	27
Eligible donor (medically suitable and officially declared brain-dead)	122
Consented donors	60
Actual donors (transported to the operating room for organ recovery)	54
Utilized donors (actual donors with at least one transplanted organ)	52
Organs/donor	3.1
